# Evolution of the Dearomative Functionalization of Activated Quinolines and Isoquinolines: Expansion of the Electrophile Scope

**DOI:** 10.1002/ange.202204682

**Published:** 2022-05-13

**Authors:** Marvin Kischkewitz, Bruno Marinic, Nicolas Kratena, Yonglin Lai, Hamish B. Hepburn, Mark Dow, Kirsten E. Christensen, Timothy J. Donohoe

**Affiliations:** ^1^ Department of Chemistry University of Oxford Chemistry Research Laboratory Mansfield Road Oxford OX1 3TA UK; ^2^ Chemical Development, Pharmaceutical Technology & Development Operations, AstraZeneca Macclesfield SK10 2NA UK

**Keywords:** Catalysis, Dearomatisation, Isoquinolines, Quinolines, Reduction

## Abstract

Herein we disclose a mild protocol for the reductive functionalisation of quinolinium and isoquinolinium salts. The reaction proceeds under transition‐metal‐free conditions as well as under rhodium catalysis with very low catalyst loadings (0.01 mol %) and uses inexpensive formic acid as the terminal reductant. A wide range of electrophiles, including enones, imides, unsaturated esters and sulfones, β‐nitro styrenes and aldehydes are intercepted by the in situ formed enamine species forming a large variety of substituted tetrahydro(iso)quinolines. Electrophiles are incorporated at the C‐3 and C‐4 position for quinolines and isoquinolines respectively, providing access to substitution patterns which are not favoured in electrophilic or nucleophilic aromatic substitution. Finally, this reactivity was exploited to facilitate three types of annulation reactions, giving rise to complex polycyclic products of a formal [3+3] or [4+2] cycloaddition.

## Graphical abstract

Reductive functionalisation of quinolinium and isoquinolinium salts with formic acid as the terminal reductant was possible under transition‐metal‐free conditions or rhodium catalysis with very low catalyst loadings. A wide range of electrophiles, including enones, imides, unsaturated esters and sulfones, β‐nitrostyrenes and aldehydes, were intercepted by the enamine species formed in situ to generate substituted tetrahydro(iso)quinolines.

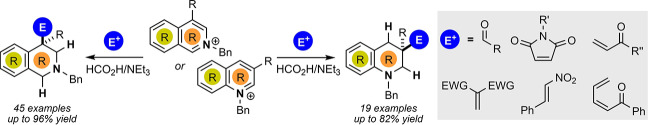

## Introduction

**Scheme 1:**
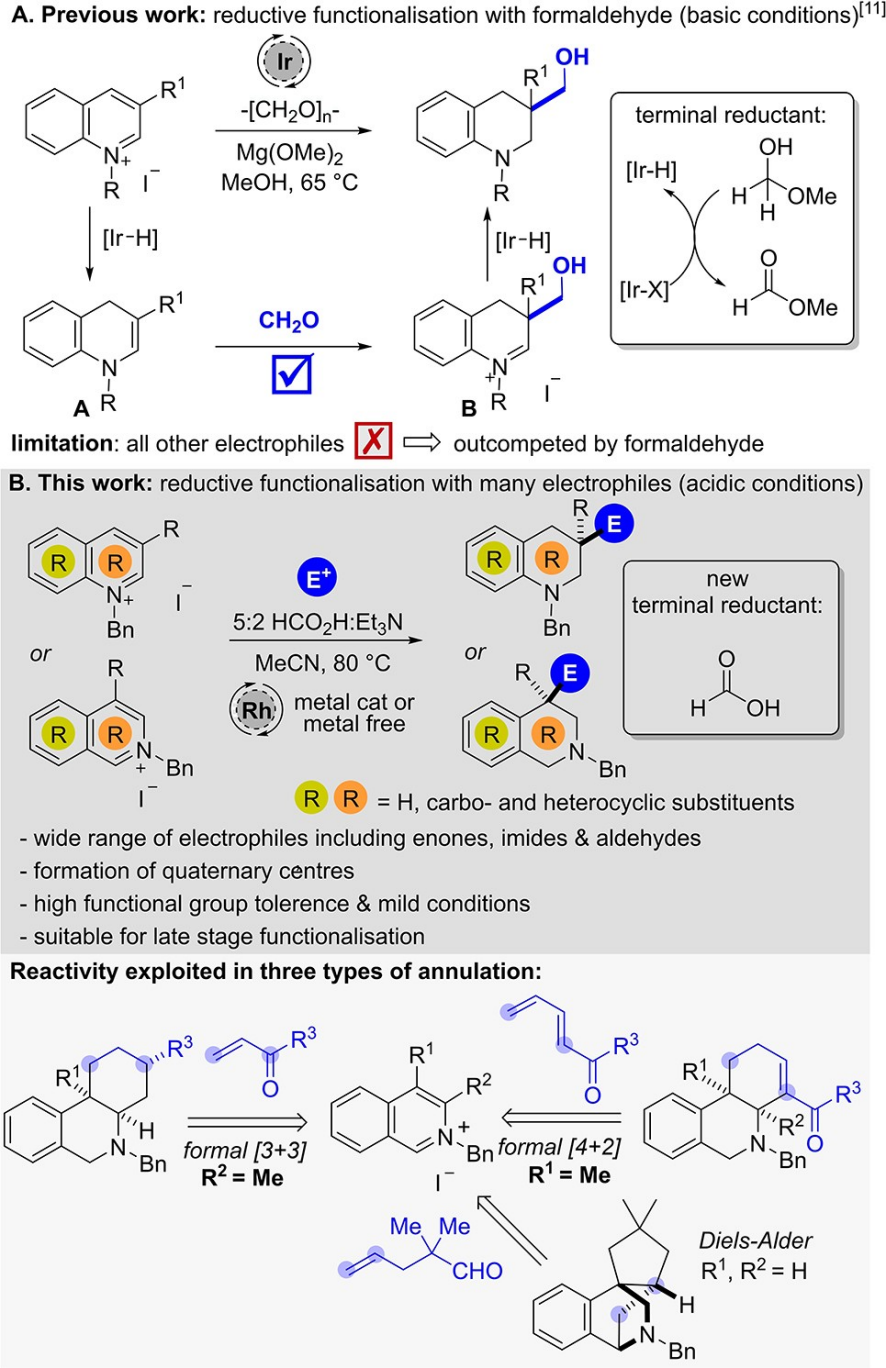
Previous work on quinolinium functionalization and a new system for quinolines and isoquinolines.

The discovery of novel methods for the synthesis of three‐dimensional nitrogen‐containing heterocycles is fundamentally important in the preparation of natural products and medicinally relevant compounds.^[1]^ Synthetic routes to aromatic nitrogen containing heterocycles have been thoroughly explored since the 19^th^ century^[2]^ and the dearomatisation of these readily available starting materials presents an attractive approach to access saturated *N*‐heterocycles.[^3^, ^4^] This synthetic strategy usually requires installation of substituents prior to the (partial) reduction process. However, cascade reactions that exploit the formation of reactive intermediates during a dearomatisation, and incorporate functionality in a single synthetic operation are substantially more attractive.^[5]^ Importantly, this approach also allows for the construction of a quaternary centre^[6]^ on the saturated heterocycle. Traditionally, such transformations have been performed stepwise with stoichiometric reductants such as borohydride reagents,^[7]^ LiAlH_4_ ^[8]^ or under Birch‐type conditions (Li/Na in ammonia^[9]^ or in the presence of biphenyl compounds^[10]^). A modern strategy avoiding the use of stoichiometric metals utilises well‐defined transition‐metal hydride complexes, which can be formed in situ in a transfer hydrogenation process. Recently, we developed a reductive hydroxymethylation reaction of activated pyridinium and quinolinium salts under iridium catalysis by harnessing formaldehyde as both an electrophile and the terminal reductant (Scheme 1A).^[11]^ Mechanistically, these reactions proceed via partial Ir−H reduction of the arene to form enamine intermediate **A**, which reacts with formaldehyde.

The resulting iminium ion **B** eventually undergoes reduction by another Ir−H species providing the desired hydroxymethylated product. The Ir−H‐species added to the ring originates from an Ir‐catalyzed oxidation of the hemi‐acetal formed in situ through the addition of methanol to formaldehyde, thus forming methyl formate. However, due to the high electrophilicity of the nascent (and essential) formaldehyde reagent no other electrophiles could be employed in this reaction, thereby significantly limiting the chemical space accessible via reductive functionalisation. Additionally, the strongly basic reaction conditions were not compatible with substrates bearing groups with acidic protons, especially C2‐alkyl substituted quinolines and C1 or C3‐alkyl substituted isoquinolines.^[12]^ As a solution, we proposed a shift from basic to acidic reaction conditions using formic acid^[13]^ instead of formaldehyde as the terminal reductant (Scheme 1B). There are two major challenges associated with this reaction design. Firstly, the reductive species formed in situ should be reactive enough to reduce the activated arene, but not directly reduce the added electrophile. Secondly, the acidity of the formic acid must be attenuated to prevent a competing direct protonation pathway of the enamine intermediate, which would lead to simple reduction without trapping of the electrophile.

## Results and Discussion

Our primary investigations were focused on the development of acidic conditions in order to expand the scope of electrophiles utilised in reductive functionalisation. We commenced our screening and optimisation experiments with the more challenging quinolinium system and C3‐methyl‐substituted quinolinium salt **1 a** was chosen as a model substrate in combination with commercially available methyl vinyl ketone (MVK) as a reactive electrophile (Table 1). Inspired by the transfer hydrogenation protocols reported by Xiao and co‐workers,^[14]^ quinolinium salt **1 a** was initially reacted with 10 equivalents of MVK in a 5 : 2 mixture of formic acid and triethylamine as hydride source and solvent in the presence of 0.01 mol % Rh‐catalyst ([RhCp*Cl_2_]_2_) and potassium iodide at 40 °C (based on the work of Xiao, iodide ligand is thought to enhance the reducing ability of metal hydrides formed in situ^[14a]^). Pleasingly, this experiment provided the desired product **2 a** in 59 % yield, along with 5 % of the simple reduced tetrahydroisoquinoline **3 a** side product (Table 1, entry 1). The formation of **3 a** results from a competing pathway in which a reactive enamine (see **A**) undergoes protonation followed by reduction. Note that in the absence of the *N*‐activating benzyl group, no desired product was observed. In an attempt to decrease the large excess of reductant and electrophile we added a solvent. Among many solvents tested (see Supporting Information) acetonitrile (0.125 M) turned out to be most suitable and delivered 41 % of the product with only 10 equivalents of reductant (entry 2). Running a control experiment without transition metal attenuated the reactivity and so we continued to investigate the effect of the temperature in the presence of 0.01 mol % of Rh‐catalyst (entry 3). A successive increase of the reaction temperature resulted in a noticeable improvement in yield to 80 % (entries 4–5). In addition to yield, we also considered the use of lower amounts of electrophile to be important, but a reduction of the MVK to 2.0 equivalents led to a dramatic drop in yield to 28 % and the formation of 50 % of side product **3 a** was observed (entry 6). In an attempt to favour the desired pathway with less electrophile, we increased the concentration by an order of magnitude, and we were pleased to find that **2 a** was obtained in an improved yield of 50 % (entry 7). A further reduction of the reductant to 4.0 equivalents and a further increase in temperature to 80 °C resulted in an additional improvement, providing **2 a** in 62 % yield (entry 8). To our surprise, and in sharp contrast to the control experiment at 40 °C (entry 3), the reaction in the absence of rhodium catalyst at 80 °C was beneficial to the yield and delivered the desired product in 74 % (entry 9). Notably, only 4 equivalents of the 5 : 2 formic acid/triethylamine complex are required, highlighting the high efficiency of this process. Finally, a small increase in electrophile loading to 5.0 equivalents (entry 10) led to a substantial increase in yield, giving **2 a** in 78 % isolated yield.

**Table 1:** Optimisation of the reaction conditions: [a] ^1^H NMR yield using trimethoxybenzene as internal standard at 0.125 mmol scale; [b] neat; [c] no metal catalyst; [d] no KI; [e] isolated yield after column chromatography.

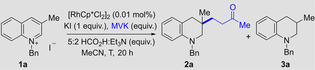					
#	MVK [equiv]	FA : NEt_3_ [equiv]	*c* [m]	*T* [°C]	Yield [%] **2 a/3 a** ^[a]^
1^[b]^	10	100	0.125	40	59/5
2	10	10	0.125	40	41/12
3^[c]^	10	10	0.125	40	15/<5
4	10	10	0.125	60	68/9
5	10	10	0.125	75	80/20
6	2	10	0.125	75	28/50
7	2	10	1.25	75	50/30
8^[d]^	2	4	1.25	80	62/35
9^[c,d]^	2	4	1.25	80	74/22
**10^[c,d]^**	**5**	**4**	**1.25**	**80**	**86 (78)^[e]^/<5**
11^[c,d]^	0	4	1.25	80	0/77^[e]^

To ensure that this transformation was truly transition‐metal free we carried out a series of control experiments with new glassware and new stirrer bars for both quinolinium and isoquinolium salts, respectively.^[15]^ In addition, the corresponding starting materials **1 a** and **4 a** (see below) were treated with a metal scavenging resin (Biotage Si‐TMT) to remove trace metal impurities and secondly new or freshly distilled reagents were used (see Supporting Information). The results of all control experiments at 80 °C delivered the desired product in comparable yields to the Rh‐catalysed reactions, strongly indicating that this transformation can also proceed without the involvement of any transition metals. Omitting the electrophile in the reaction provided product **3 a** in good yield as predicted (entry 11). The Supporting Information contains details of a full optimisation study examining many reaction parameters (e.g., the ratio of HCO_2_H to Et_3_N) and these also show a clear correlation between reaction temperature and conversion. The observed reactivity is also supported by precedent in the literature, in which formic acid is used in the transition‐metal free reduction of imines at elevated temperatures.^[16]^ The most prominent of such reactions is the Wallach reaction,^[17]^ which converts aldehydes or ketones to amines by a reductive amination in the presence of heat and formic acid. Similar reactivity was noted in the Leuckart reaction, in which the ammonium salts of formic acid or formamide are used.^[18]^

**Scheme 2:**
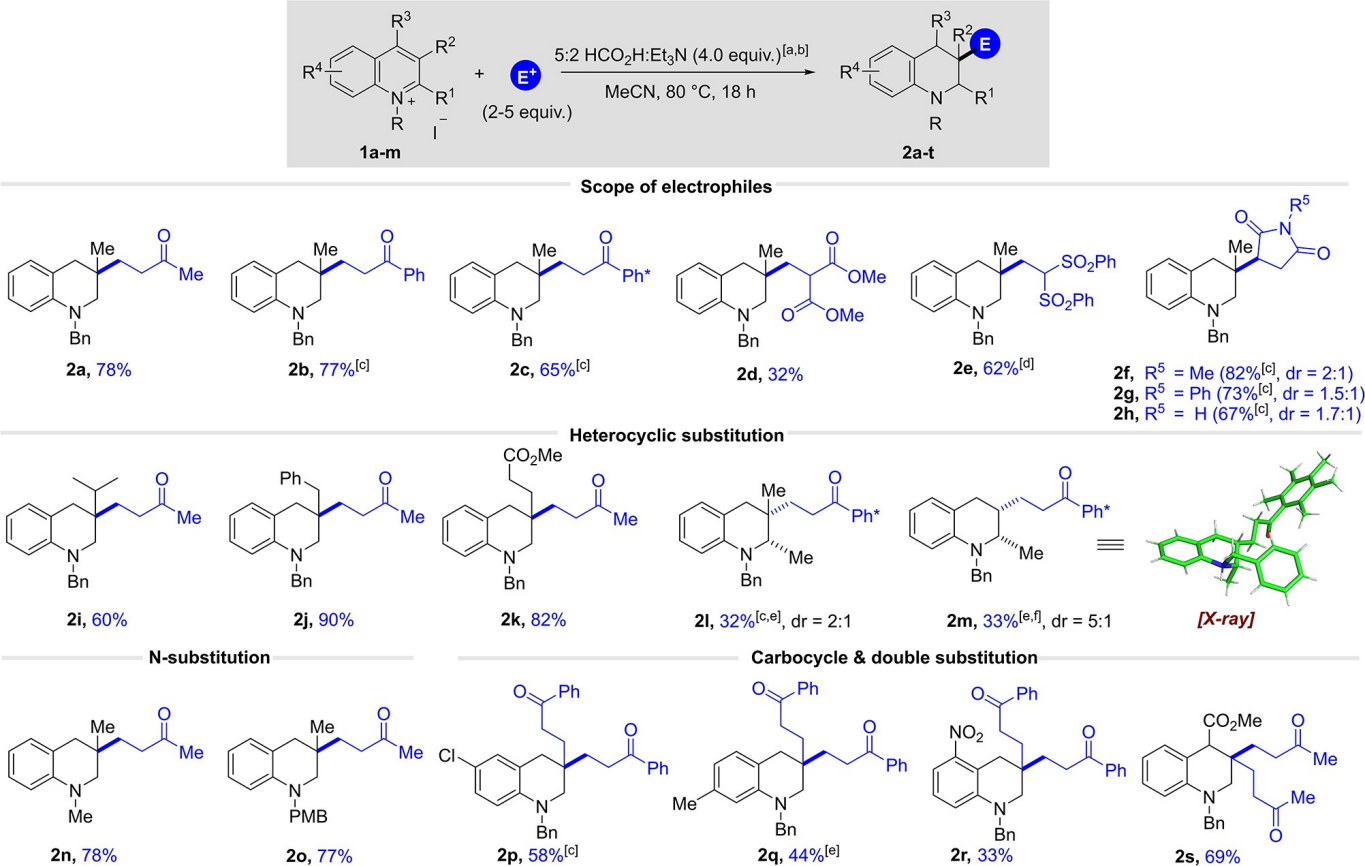
Substrate scope for the synthesis of functionalized tetrahydroquinolines. [a] Reaction conditions: quinolinium salt **1** (1.0 equiv), electrophile (5.0 equiv), 5 : 2 HCO_2_H : Et_3_N (4.0 equiv), MeCN (1.25 M), 80 °C, 18 h. [b] Yields refer to isolated material after column chromatography. [c] Reaction conducted with 2.0 equiv of electrophile. [d] Reaction conducted with 4.0 equiv of electrophile and 8.0 equiv of 5 : 2 HCO_2_H : Et_3_N. [e] Reaction conducted in the presence of 0.01 mol % [RhCp*Cl_2_]_2_ at 80 °C. [f] Reaction conducted with 1.5 equiv of electrophile. The relative stereochemistry of **2 f**–**2 h** is unassigned.

With optimised conditions in hand (with catalyst, see entry 8; metal free, see entry 10), we set out to explore the scope of electrophiles and substrates that could be employed in this reductive functionalisation reaction. While primarily focusing on the optimised transition metal free conditions, in certain cases of more challenging (highly substituted) arene substrates or for electrophiles with low boiling points, the additional use of 0.01 mol % Rh‐catalyst was employed because it increased the yield of the reaction; see below for a study of the differences in rates when the two sets of conditions are employed. The Supporting Information contains details of yields for several preparative reactions in the presence and absence of metal catalyst. Thus, the substrate **1 a** from the optimisation experiments was reacted with a range of 1,4‐acceptor electrophiles (Scheme 2). A range of enones including methyl‐, phenyl‐ and pentamethylphenyl (Ph*)^[19]^ vinyl ketone gave the desired products (**2 a** to **2 c**) in good yields. Methyl acrylate was not electrophilic enough to be trapped by the enamine intermediate, however the more reactive 2‐methylenemalonate gave the desired product (**2 d**) in lower yield (32 %). Similarly an α,β‐unsaturated disulfone could be incorporated in 62 % yield (**2 e**). Finally, the reaction proceeded in good yields with a range maleimides to give the tetrahydroquinolines (**2 f** to **2 h**), with moderate diastereoselectivity. The Supporting Information contains a list of electrophiles that were not able to successfully trap the enamine intermediate. Next, variation of the substituent in the quinoline C3 position was examined. Using MVK as the electrophile, bulky isopropyl (**2 i**, 60 %), benzyl (**2 j**, 90 %) and propanoate groups (**2 k**, 82 %) were well tolerated and gave the desired products. Substitution on C2 and C4 of the quinoline core (**2 l**, **2 m** and **2 s**) was also tolerated. Note in the case of **2 m** a crystal structure of the **2 m**⋅HCl salt was obtained to confirm the stereochemical assignment^[20]^ and for **2 l** the assignment was made by analogy. Pleasingly, changing the activating group on the nitrogen to a PMB or methyl gave virtually identical results to the previously used benzyl activated quinoliniums (**2 n** and **2 o**). The lower yields of **2 m** (which lacks a C3 substituent on the starting quinoline) were caused by a competing pathway of simple reduction and double substitution at C3 by another equivalent of electrophile. Indeed, we could harness this reactivity as demonstrated with the formation of **2 p** to **2 s** from quinolines lacking a C3 substituent. Furthermore, substitution on the aromatic portion of the quinolinium had no profound influence on the reactivity, as expected, and the double substituted products **2 p** to **2 r** were isolated in yields ranging from 33 % to 58 %.

**Scheme 3:**
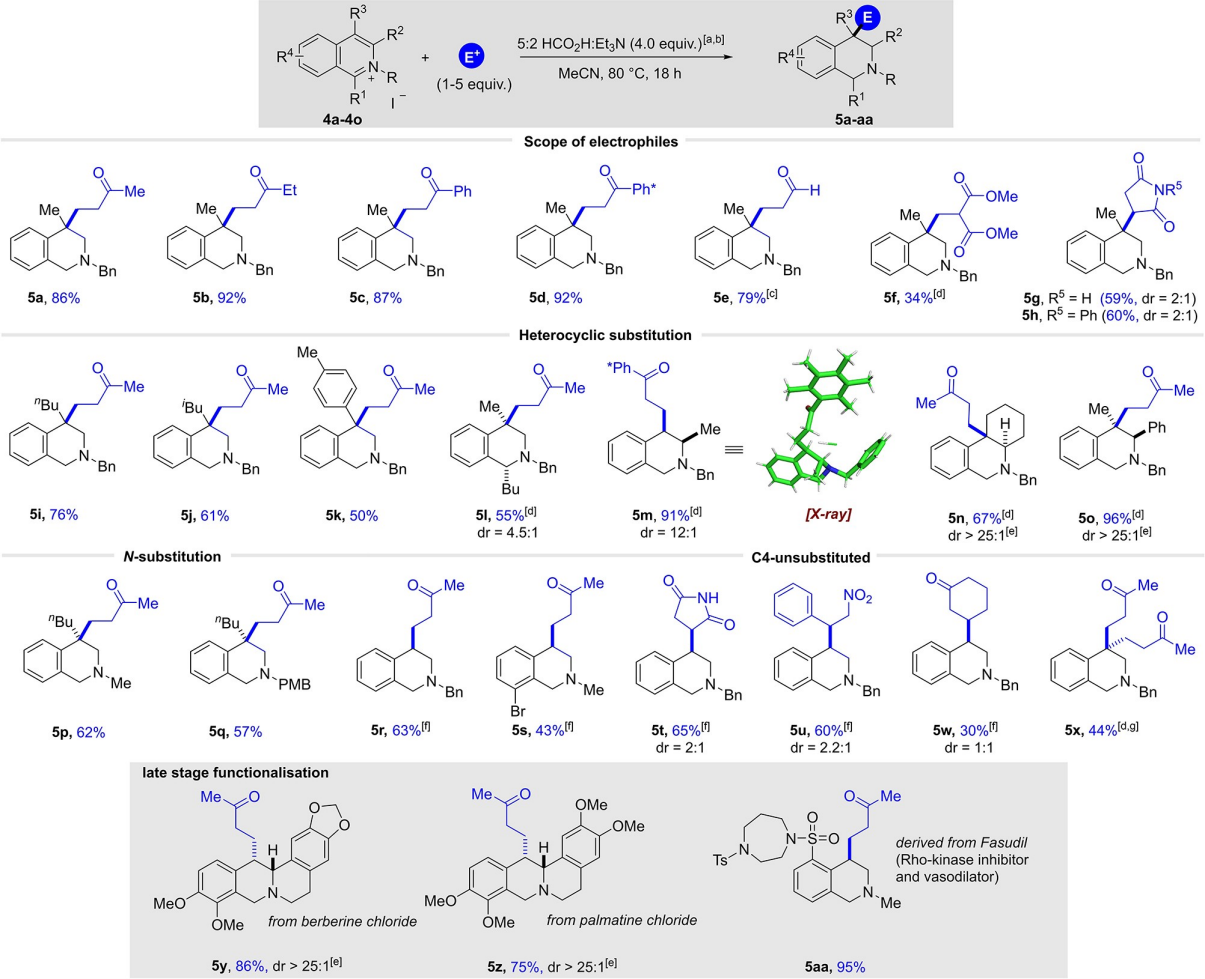
Substrate scope for the synthesis of functionalized tetrahydroisoquinolines: [a] Reaction conditions: isoquinolinium salt **4** (1.0 equiv), electrophile (2.0 equiv), 5 : 2 HCO_2_H : Et_3_N (4.0 equiv), MeCN (1.25 M), 80 °C, 18 h. [b] Yields refer to isolated material after column chromatography. [c] Reaction conducted in the presence of 0.01 mol % [RhCp*Cl_2_]_2_ at 50 °C. [d] Reaction conducted in the presence of 0.01 mol % [RhCp*Cl_2_]_2_ at 75 °C. [e] For compound with dr>25 : 1 only one diastereomer was detectable in the crude ^1^H NMR spectrum. [f] Reaction conducted with 1.0 equiv of electrophile. [g] Reaction conducted with 5.0 equiv of electrophile. The relative stereochemistry of **5 g**, **5 h**, and **5 t**–**w** is unassigned. The relative stereochemistry of compounds **5 l**–**o** were assigned by NOESY experiments and **5 y**–**z** by *J*‐coupling (see Supporting Information).

With a general method for the synthesis of functionalised tetrahydroquinolines in hand we next turned our attention to the reactions of pyridinium salts. However, this proved to be substantially more challenging, and complex mixtures of products were obtained consistently; these mixtures contained compounds that were both aromatic and dearomatized, and they had incorporated between zero and two molecules of MVK electrophile. We think that one of the problems to be overcome with the reaction of pyridinium salts lies in the difficulty in dearomatizing (by hydride addition) a monocyclic arene substrate. Therefore, we moved to the preparation of tetrahydroisoquinolines, another important class of *N*‐heterocycle which proceeded smoothly without the need for re‐optimisation. Thus, the transformation of the C4‐methyl substituted isoquinolinium iodide **4 a** in combination with 2.0 equivalents of MVK (no catalyst added) was found to work with even greater efficiency and the desired tetrahydroisoquinoline **5 a** was isolated in 86 % yield (see Scheme 3). A comparable result was achieved for the corresponding bromide salt **4 a′** (83 %‐ not shown). Using the same **4 a** substrate, we next demonstrated that other enones including ethyl vinyl ketone, phenyl vinyl ketone and Ph* vinyl ketone performed very well in this reaction (**5 b** to **5 d**). Notably, this method allows the efficient introduction of an unprotected aldehyde when acrolein was used as the electrophile (**5 e**, 79 %). Due the low boiling point of acrolein (53 °C) the reaction was carried out at 50 °C which required the addition of 0.01 mol % of Rh‐catalyst to ensure reactivity. This result highlights one advantage of the alternative metal‐catalysed protocol. In analogy to the quinolinium system, the use of methyl acrylate as electrophile was not successful and only led to the formation of the reduced side product. However, the more electrophilic 2‐methylenemalonate congener delivered the desired product **5 f**, albeit in a lower yield (34 %). Moreover, the reaction proceeded efficiently with different maleimides, affording the products **5 g** and **5 h** in 59 % and 60 % yield, respectively.

Using MVK as the electrophile, we next investigated the effect of different substituents on the C4‐position of the isoquinoline and found that butyl, isobutyl and even aryl substituents are well tolerated, as documented by the successful preparation of products **5 i** to **5 k** in 76 to 50 % yield. Of note, the sterically encumbered C4‐isobutyl and *p*‐tolyl substituted substrates were not suitable for the previous basic hydroxymethylation reaction conditions yet here they gave **5 j** and **5 k** in reasonable yield.^[12]^ Encouraged by these results we next set out to examine a series of more challenging C1 and C3 alkyl‐substituted arenes. To our delight, a C1 & C4 disubstituted substrate **4 b** underwent the desired reaction to generate isoquinoline **5 l** in 55 % yield as a 4.5 : 1 ratio of diastereomers. Again, in this case the presence of Rh‐catalyst turned out to be crucial for the reactivity as only traces of product were observed without catalyst at 80 °C.

We next showed that the reaction also proceeded smoothly for a C3‐methyl substituted substrate when Ph* vinyl ketone was used as the electrophile and product **5 m** ^[20]^ was obtained in 91 % yield and diastereoselectivity of 12 : 1. The relative stereochemistry of **5 m**⋅HCl was proven by X‐ray crystallography. Finally, two substrates featuring 3,4‐disubstituion patterns were successfully tested, furnishing products **5 n** and **5 o** in good yields and d.r.<25 : 1. In analogy to the quinoline system, substitution on the nitrogen atom was not limited to a benzyl, and other protecting groups such as methyl and *para*‐methoxybenzyl gave the desired products **5 p** and **5 q** in good yields.

We then moved to the challenging C4‐unsubstituted isoquinolinium substrates. We were pleased to find that undesired double addition could be mostly prevented by simply reducing the amount of electrophile to 1.0 equivalent and both MVK and maleimide delivered the monosubstituted products **5 r**–**t** in moderate to good yields. Encouraged by these results we tested sterically encumbered electrophiles which were not suitable partners for C4‐substituted substrates. Pleasingly, we found that both β‐nitrostyrene and cyclohexenone underwent the desired transformation to give **5 u** and **5 w**, thereby further expanding the scope of electrophiles and removing the requirement for C4 substitution on the starting arene. Furthermore, if we increased the electrophile equivalents, we could access the difunctionalised product **5 x** in 44 %, which is consistent with the reactivity displayed in the quinoline series.

In order to demonstrate the utility of this methodology a series of late‐stage functionalisation reactions were performed on biologically relevant compounds. Firstly, two natural occurring alkaloids berberine and palmatine chloride could be successfully employed and the desired products **5 y** and **5 z** were isolated in high yields as single diastereomers. Furthermore, we sought to apply the reductive functionalisation to *Fasudil*, a protein kinase inhibitor that is already approved for use in Japan.^[21]^ After a sequence of tosyl protection and quaternization of the arene nitrogen the key transformation using MVK proceeded smoothly and afforded the desired product **5 aa** in an excellent yield of 95 %.

**Scheme 4:**
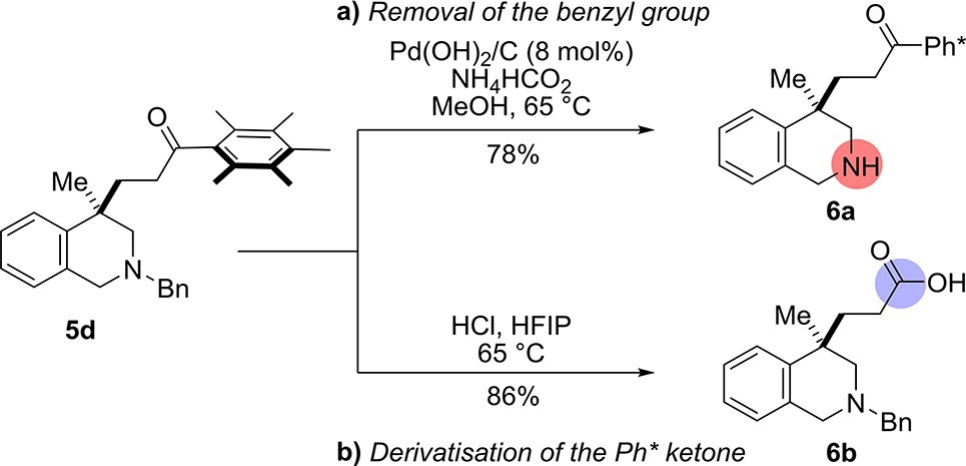
Derivatization of the tetrahydroisoquinoline products.

With the substrate scope completed, our attention turned to derivatization of the reaction products. To this end, the benzyl protecting group of the tetrahydroisoquinoline product **5 d** was removed using ammonium formate as the hydrogen transfer agent to give the free tetrahydroisoquinoline product **6 a** in 78 % yield (Scheme 4a).^[22]^ Pleasingly, our recently disclosed conditions^[23]^ for the conversion of Ph* ketones to carboxylic acids (2 M HCl in HFIP at 65 °C) proved effective in cleaving tetrahydroisoquinoline product **5 d** to give the corresponding carboxylic acid **6 b** in 86 % yield (Scheme 4b). Of note, this latter follow‐up sequence allows access to C4‐substituted tetrahydroisoquinolines (THIQs), which could not be accessed via the direct dearomative functionalisation with acrylate esters as they were not electrophilic enough to participate in the reaction.

**Scheme 5:**
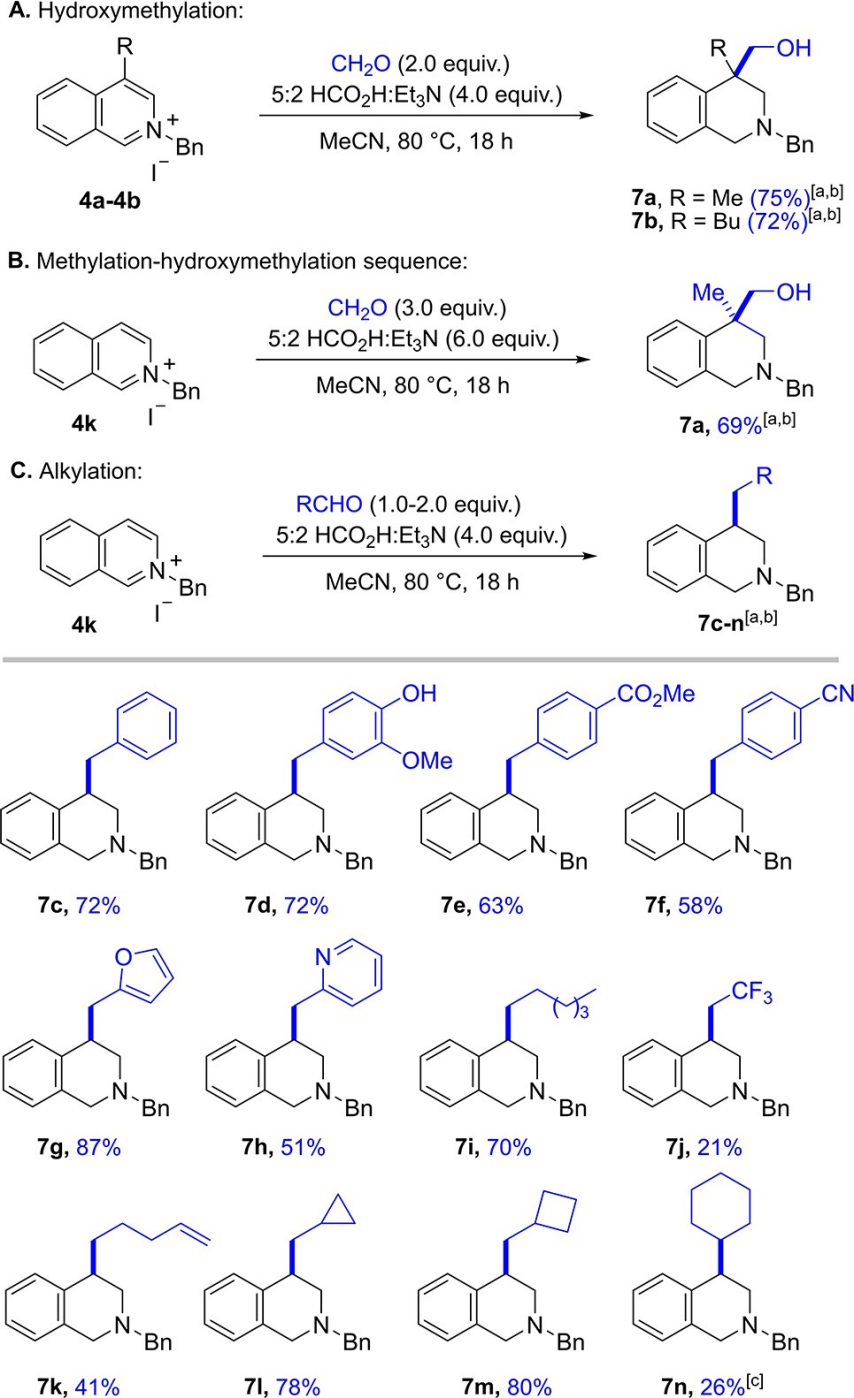
Reductive functionalization with aldehydes. A) Hydroxymethylation of 4‐substituted isoquinolinium salts with formaldehyde. B) Methylation‐hydroxymethylation cascade of unsubstituted isoquinolinium salts. C) Reductive alkylation of unsubstituted isoquinolinium salts with aldehydes: [a] Reaction conditions: isoquinolinium salt **4** (1.0 equiv), electrophile (2.0 equiv), 5 : 2 HCO_2_H : Et_3_N (4.0 equiv), MeCN (1.25 m), 80 °C, 18 h. [b] Yields refer to isolated material after column chromatography. [c] Reaction conducted in the presence of 0.01 mol % [RhCp*Cl_2_]_2_ at 80 °C.

Having explored the scope of electrophiles while focusing on conjugate additions we next turned our attention to carbonyl addition (Scheme 5).^[7c]^ Following our previous studies, we initiated our investigation by reacting two C4‐substiuted isoquinolinium salts **4 a** and **4 b** with 2.0 equivalents of aqueous formaldehyde solution under the new optimised transition metal free reaction conditions (Scheme 5A).^[12]^ Pleasingly, both transformations proceeded smoothly and the desired hydroxymethylated products **7 a** and **7 b** were isolated in 75 % and 72 % yield, respectively. Due to the high reactivity of formaldehyde, C4‐unsubstituted isoquinolinium salts were found to undergo a tandem methylation‐hydroxymethylation sequence^[24]^ leading to the formation of two new C−C bonds in one pot (this product was formed to some degree even when only 1.0 equivalent of formaldehyde was used). Hence, isoquinolinium salt **4 k** was reacted with 3.0 equivalents of formaldehyde to give 69 % of THIQ **7 a** bearing a quaternary centre (Scheme 5B).

To harvest the potential of the methodology, we were intrigued as to whether the acidic conditions would tolerate the use of other aldehydes. Pleasingly, when using 1 equivalent of the less reactive benzaldehyde as an electrophile the isoquinolinium **4 k** underwent mono functionalisation to give product **7 c** in 72 % yield, thereby representing a useful method for the reductive alkylation of unsubstituted isoquinolinium salts. The lack of a hydroxyl group in the product is noteworthy; the alkylation likely proceeds via an initial attack at the carbonyl by the enamine, followed by elimination of the newly formed hydroxyl. The resulting α,β‐unsaturated iminium species can then be reduced by a hydride source to deliver the product.

Encouraged by this result we evaluated the generality of this process. We found that benzaldehydes bearing nitrile, ester or phenol functionalities were tolerated, delivering products **7 d** to **7 f** in good yields (Scheme 5C). We examined heteroaromatic aldehydes and were pleased to find that both furfural and picolinaldehyde behaved well and products **7 g** and **7 h** were isolated in 87 % and 51 % yield, respectively. In addition, aliphatic aldehydes such as hexanal could be employed (**7 i**) and the use 2,2,2,‐trifluoroacetaldehyde allowed the introduction of a trifluoromethyl group **7 j**, albeit in lower yield. Notably, isolated olefins are tolerated under the reaction conditions to furnish **7 k** in 41 % yield. The use of different formyl substituted cycloalkanes allowed the efficient introduction of a cyclopropane and a cyclobutane moiety (**7 l** and **7 m**). Finally, even the alkylation of a ketone was feasible, as demonstrated when cyclohexanone was employed as the electrophile, giving cyclohexyl substituted THIQ **7 n** in 26 % yield. In this case, the addition of 0.01 mol % of rhodium catalyst was required, presumably to reduce the substituted unsaturated iminium salt.

**Scheme 6:**
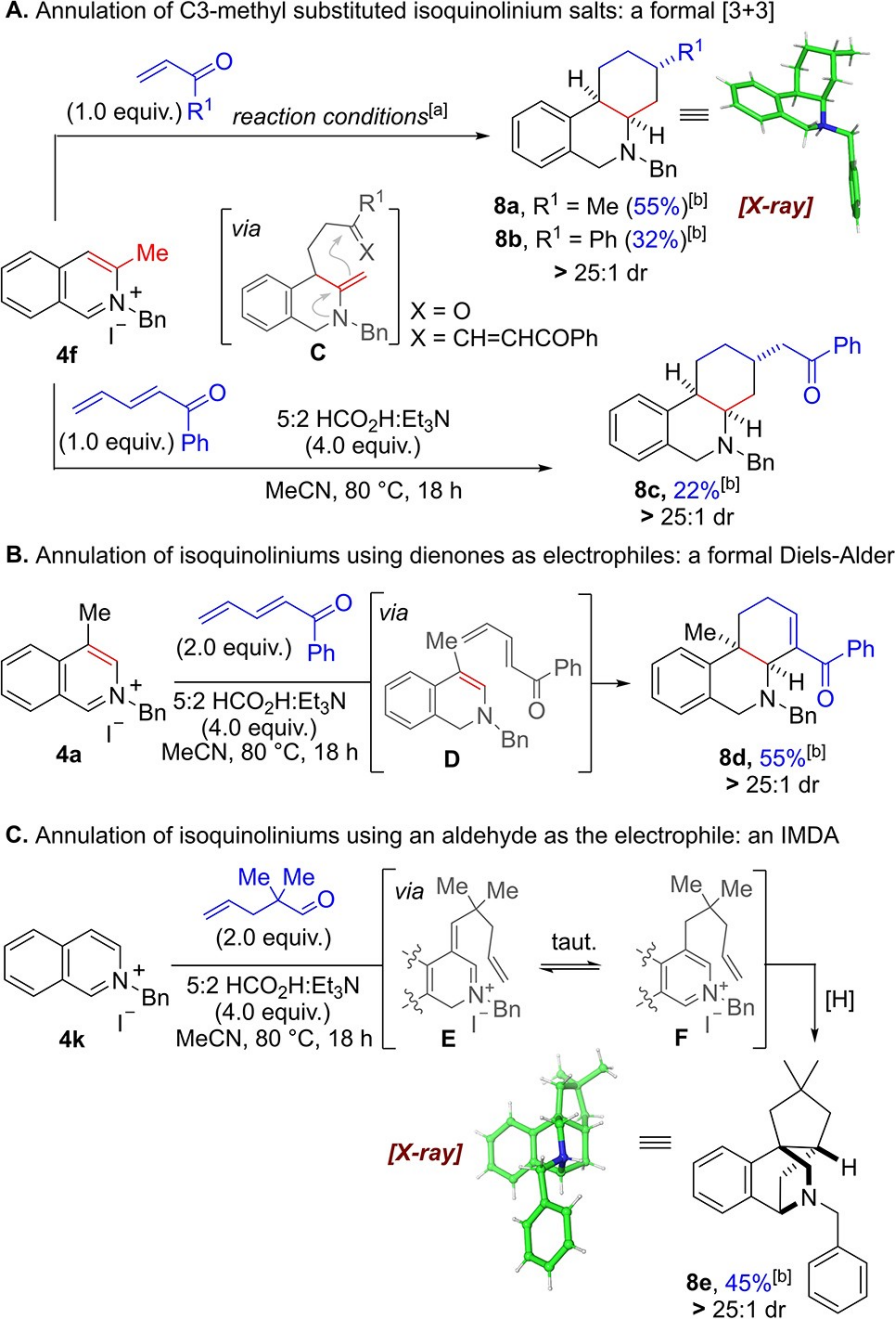
Annulation of isoquinolinium salts. [a] Reaction conditions: isoquinolinium salt **4** (1.0 equiv), electrophile (1.0 equiv), 5 : 2 HCO_2_H : Et_3_N (4.0 equiv), 0.01 mol % [RhCp*Cl_2_]_2_ MeCN (1.25 M), 80 °C, 18 h. [b] Yields refer to isolated material after column chromatography.

At this point we strived to exploit this new methodology for annulation processes in order to make more complex polycyclic structures (Scheme 6). In the first approach the C3‐methyl isoquinolinium salt **4 f** was reacted with 1.0 equivalent of an enone in the presence of 0.01 mol % of Rh‐catalyst (Scheme 6A). Pleasingly, this transformation delivered the annulation products **8 a**,**b** as single diastereomers. The relative stereochemistry was unambiguously assigned by single crystal X‐ray crystallographic analysis of **8 a**⋅HCl^[20]^ and NOE experiments (see Supporting Information).

Mechanistically, this formal [3+3] cycloaddition likely proceeds stepwise via initial C1‐reduction of the substrate followed by trapping of the enamine at the C4‐position. By utilising the C3‐methyl group the resulting iminium adduct tautomerises to the less substituted exocyclic enamine (see intermediate **C**), which in turn attacks the carbonyl group intramolecularly (6‐*exo*‐trig). Hence, the rate of exocyclic enamine formation and the ensuing attack onto the carbonyl group must be higher than the rate of iminium ion reduction. Under weakly acidic conditions the resulting alcohol intermediate undergoes elimination to form an α,β‐unsaturated iminium species, which is finally reduced (twice) in a highly diastereoselective fashion. This reactivity was further expanded to encompass a dienone electrophile, to furnish annulated product **8 c**, albeit in lower yield. In this case the corresponding enamine 1,6‐addition adduct initially isomerises to a conjugated enone, which subsequently undergoes an intramolecular 1,4‐addition (6‐*exo*‐trig) followed by reduction.

Interestingly, when moving the methyl group substituent to the C4‐postion the annulation reactivity can be altered to a formal Diels–Alder reaction, presumably on an enamine intermediate, delivering annulated *cis*‐product **8 d** as single diastereomer in 55 % yield (Scheme 6B). This process can conceivably proceed through a stepwise mechanism, by 1,6‐addition of the enamine **D** followed by intramolecular attack of the enol onto the iminium, or in a concerted fashion through an inverse electron‐demand Diels–Alder reaction. A final isomerisation of the remaining double bond delivers a stable enone, which in the absence of Rh‐catalyst is not reduced, thereby allowing further functionalisation.

Finally, when substituted aldehydes such as 2,2‐dimethylpentenal were used in the metal free alkylation, a cascade of reductive alkylation/Diels–Alder type reactivity was observed, delivering tetracycle **8 e** directly in 45 % yield as a single diastereomer (Scheme 6C). This reaction likely proceeds through enamine alkylation of the aldehyde, giving rise to α,β‐unsaturated iminium **E**; it is reasonable to assume that conjugate reduction of **E** is relatively slow because of impedance provided by the two methyl groups. Therefore, **E** can conceivably tautomerise to isoquinolinium **F** capable of undergoing an intramolecular formal [4+2] cycloaddition.^[25]^ A final reduction of the newly formed iminium furnishes the product. The relative configuration of **8 e** ^[20]^ was again determined by single crystal X‐ray diffraction and NOE experiments (see Supporting Information). Interestingly, example **7 k** (Scheme 5, see above) representing the same type of alkylation but without the *gem*‐dimethyl group next to the carbonyl oxygen did not provide the annulation product in significant quantities, also suggesting that a Thorpe–Ingold effect is responsible for a rapid cycloaddition.

**Scheme 7:**
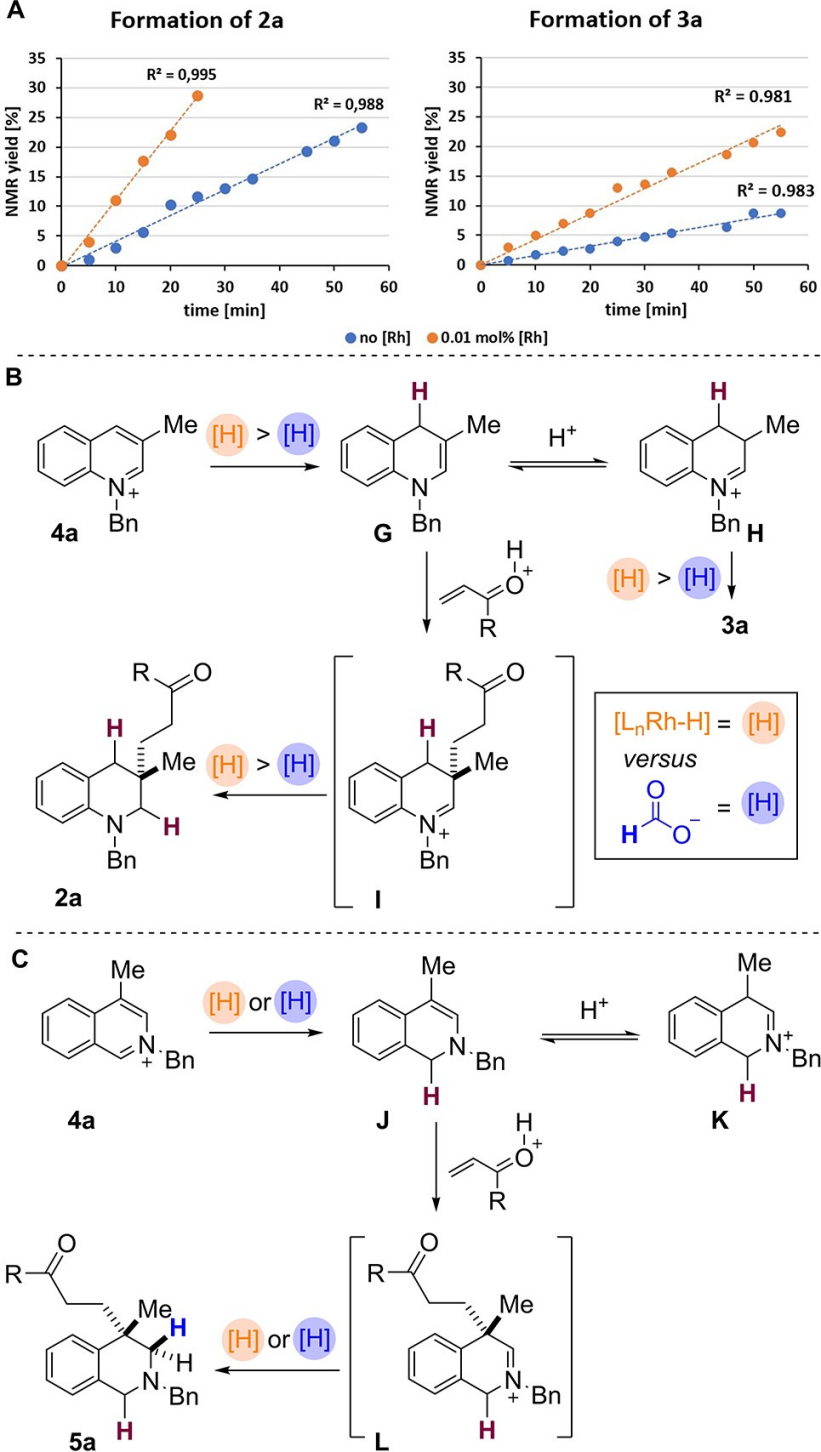
A) Kinetic profile of the quinoline reaction. B) Proposed mechanism for the quinolinium salts. C) Proposed mechanism for the reduction of isoquinolinium salts.

Finally, to explore the differences between the transition‐metal free and the metal catalysed pathway, the kinetic profile of the reaction of quinolinium **4 a** was investigated (Scheme 7A). Remarkably, even a low catalyst loading of 0.01 mol % [RhCp*Cl_2_]_2_ resulted in a significant increase in the initial reaction rate. Scheme 7A shows relative formation of the desired product **2 a** under the two different sets of conditions; note that the rates of both reactions then levelled off so that similar final conversions to **2 a** were observed after 5–6 h (see Supporting Information).

It is interesting to note that the initial rate of formation of both **2 a** *and* side‐product **3 a** was approximately 2.5 times faster in the presence of rhodium catalyst, compared to the metal‐free conditions. This data also shows that under metal catalysed conditions the amount of the side‐product **3 a** formed in the reaction is higher than that formed under the metal‐free conditions (Scheme 7A; for confirmation see also Table 1).

We suggest that this data can most likely be attributed to the different reducing species present in the two sets of conditions; under rhodium catalysis a metal hydride [e.g. cp*L_*n*_Rh−H] is postulated, versus the presence of formate ion in the metal‐free conditions. A more effective rhodium hydride reducing agent is likely to lead to a faster reduction of the starting arene to form **G** (and therefore faster formation of the product **2 a** via **I**, Scheme 7B). However, if the enamine intermediate **G** is in rapid equilibrium with iminium **H** then this would also be reduced more rapidly in the presence of a metal catalyst, thus skewing the aforementioned equilibrium and producing more of the side‐product **3 a**, as observed.

As an extension, we propose a similar mechanism for isoquinolinium reduction, which again takes place in the presence (or absence) of rhodium catalysis (Scheme 7C). Initially, hydride adds to the C1‐position of **4 a**. The resulting enamine **J** subsequently undergoes a 1,4‐addition to (for example) a vinyl ketone generating iminium species **L**. In the final reduction step hydride is then added to iminium **L** to form the product **5 a**. Given the similarity between the mechanisms for the two arenes, we predict that the enamine **J** is in rapid equilibrium with iminium **K**. Note that in some cases we did observe small amounts of products derived from the reduction of **K**.

**Scheme 8:**
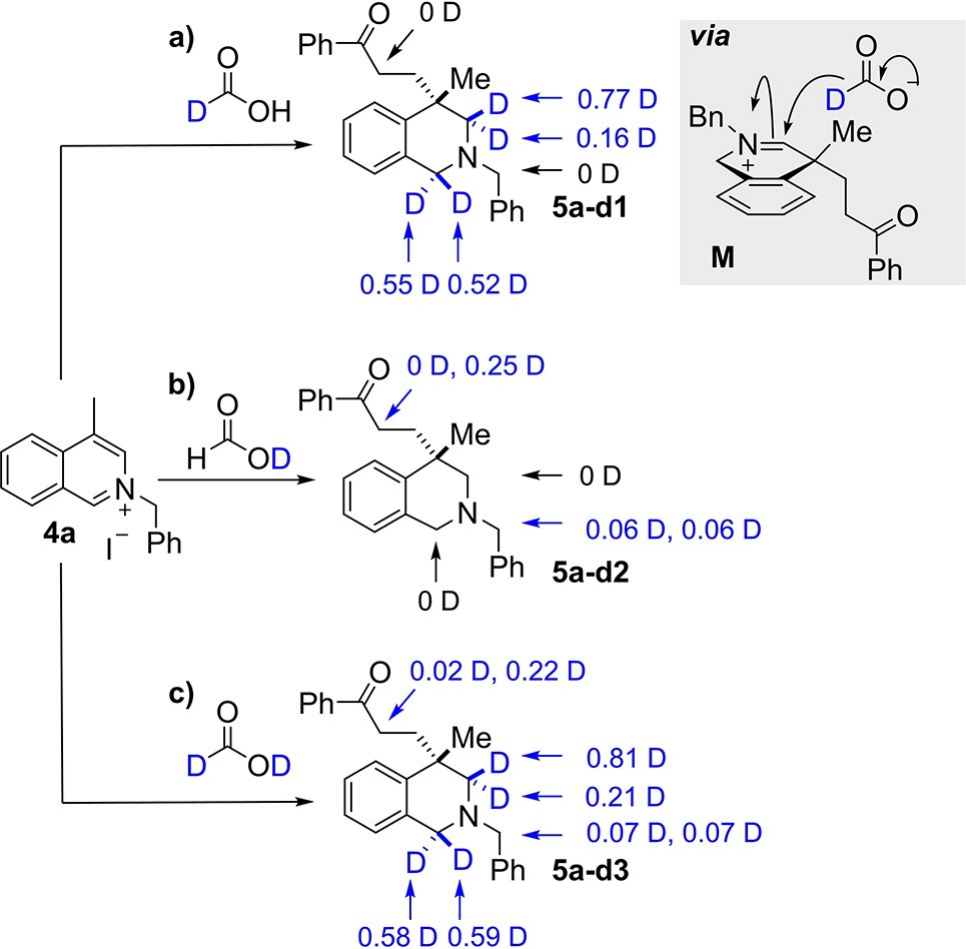
Deuterium‐labelling studies on the reaction of an isoquinolinium salt.

To shed more light on the general mechanism of dearomatisation, nucleophilic attack and then reduction, a series of deuterium labelling experiments were undertaken on both quinoline and isoquinoline substrates. At first, model isoquinoline substrate **4 a** was subjected to the optimised conditions with phenyl vinyl ketone as the electrophile and three appropriately deuterium‐labelled reagents (Scheme 8). Firstly, the use of formic‐d1 acid (DCO_2_H) resulted in the formation of **5 a‐d1** with one atom of deuterium incorporated at the C1‐ and C3‐position, respectively (Scheme 8A, path a). No diastereoselectivity was observed for deuterium incorporation into the C1‐position (0.55D vs 0.52D); this is as expected because no selectivity would originate from addition to the starting arene **4 a**. Interestingly, deuterium incorporation at the C3‐position did show a facial selectivity of approximately 4 : 1 (0.77D vs 0.16D). NOESY analysis of parent compound **5 a‐d1** confirmed that most of the deuterium incorporation (0.77D) had occurred *syn* to the methyl substituent, presumably because the second hydride is delivered to the less hindered face of the iminium ion (see **M**). A second experiment with formic acid‐d1 (HCO_2_D) led to the formation of **5 a‐d2** with no deuterium incorporated into the C1‐ or C3‐position (Scheme 8, path b). However, deuterium incorporation was observed at the acidic *N*‐benzyl position (0.12D) and the α‐carbonyl position (0.25D). We hypothesized that the incorporation of deuterium at the latter position results from deuterium trapping of the enol intermediate after the initial 1,4‐addition, but were puzzled by the low D incorporation at this position. However, quenching a repeat reaction with D_2_O then resulted in a significantly higher incorporation at the α‐carbonyl position (see Supporting Information). This implicates an exchange process that takes place during work‐up and can explain the results. Finally, using formic acid‐d2 (DCO_2_D) led to a cumulative effect of the variations discussed above (Scheme 8, path c, **5 a‐d3**).

Note that the same levels of deuterium incorporation were observed when [RhCp*Cl_2_]_2_ was used as a catalyst in these labelling experiments. Moreover, a congruent outcome with respect to D incorporation was obtained for the corresponding deuterium labelling experiments using quinolinium salt **1 a** (see Supporting Information for details). Thus, the labelling experiments support the mechanisms postulated in Scheme 7.

## Conclusion

In conclusion the scope of electrophiles for the reductive functionalisation of both quinolinium and isoquinolinium salts has been greatly expanded. This was achieved by switching to mild acidic conditions using the formic acid‐triethylamine 5 : 2 complex as the terminal reductant. A wide range of Michael‐acceptors (ketones, imides, sulfones, malonates) as well as aldehydes were tolerated and gave the C3/C4 functionalised tetrahydro(iso)quinolines in good yields, in most cases without the involvement of a transition metal catalyst. Moreover, a variety of substituents and substitution patterns on the parent (iso)quinolinium substrate were tolerated leading to the stereoselective synthesis of complex ring structures. We could further harness this reactivity in three different annulation reactions providing complex tri‐ and tetracyclic frameworks in a single step. Additionally, cleavage of the activating group on the nitrogen, as well as of the pentamethylphenyl group was demonstrated. Expansion of this novel reactivity to pyridines and the application for the synthesis of bioactive molecules and/or natural products and the development of an asymmetric version are ongoing in our laboratories and will be reported in due course.

## Conflict of interest

The authors declare no conflicts of interest.

1

## Supporting information

As a service to our authors and readers, this journal provides supporting information supplied by the authors. Such materials are peer reviewed and may be re‐organized for online delivery, but are not copy‐edited or typeset. Technical support issues arising from supporting information (other than missing files) should be addressed to the authors.

Supporting Information

## Data Availability

The data that support the findings of this study are available in the Supporting Information of this article.
